# Correlation between global methylation level of peripheral blood leukocytes and serum C reactive protein level modified by MTHFR polymorphism: a cross-sectional study

**DOI:** 10.1186/s12885-018-4089-z

**Published:** 2018-02-13

**Authors:** Masanori Nojima, Motoki Iwasaki, Yoshio Kasuga, Shiro Yokoyama, Hiroshi Onuma, Hideki Nishimura, Ritsu Kusama, Teruhiko Yoshida, Shoichiro Tsugane

**Affiliations:** 1 Center for Translational Research, The Institute of Medical Science Hospital, The University of Tokyo, 4-6-1 Shirokanedai, Minato-ku, Tokyo, 108-8639 Japan; 2 Division of Epidemiology, Research Center for Cancer Prevention and Screening, National Cancer Center, 5-1-1 Tsukiji, Chuo-ku, Tokyo, 104-0045 Japan; 3 Department of Surgery, Nagano Matsushiro General Hospital, 183 Matsushiro, Matsushiro-cho, Nagano City, Nagano Prefecture 381-1231 Japan; 4 Department of Breast and Thyroid Surgery, Nagano Red Cross Hospital, 5-22-1 Wakasato, Nagano City, Nagano Prefecture 380-8582 Japan; 5 Department of Respiratory Surgery and Breast Surgery, Nagano Municipal Hospital, 1333-1 Tomitake, Nagano City, Nagano Prefecture 381-8551 Japan; 6 Department of Surgery, Hokushin General Hospital, 1-5-63 Nishi, Nakano City, Nagano Prefecture 383-8505 Japan; 7 Division of Genetics, National Cancer Center Research Institute, 5-1-1 Tsukiji, Chuo-ku, Tokyo, 104-0045 Japan; 8 Research Center for Cancer Prevention and Screening, National Cancer Center, 5-1-1 Tsukiji, Chuo-ku, Tokyo, 104-0045 Japan

**Keywords:** DNA methylation, C reactive protein, Inflammation, Folate metabolism, Folate intake

## Abstract

**Background:**

Chronic inflammatory conditions are associated with higher tumor incidence through epigenetic and genetic alterations. Here, we focused on an association between an inflammation marker, C-reactive-protein (CRP), and global DNA methylation levels of peripheral blood leukocytes.

**Methods:**

The subjects were 384 healthy Japanese women enrolled as the control group of a case-control study for breast cancer conducted from 2001 to 2005. Global DNA methylation was quantified by Luminometric Methylation Assay (LUMA).

**Results:**

With adjustment for lifestyle-related factors, including folate intake, the global DNA methylation level of peripheral blood leukocytes was significantly but weakly increased by 0.43% per quartile category for CRP (*P* for trend = 0.010). Estimated methylation levels stratified by CRP quartile were 70.0%, 70.8%, 71.4%, and 71.3%, respectively. In addition, interaction between polymorphism of MTHFR (rs1801133, known as C677T) and CRP was significant (*P* for interaction = 0.046); the global methylation level was significantly increased by 0.61% per quartile category for CRP in the CT/TT group (those with the minor allele T, *P* for trend = 0.001), whereas no association was observed in the CC group (wild type).

**Conclusions:**

Our study suggests that CRP concentration is weakly associated with global DNA methylation level. However, this association was observed more clearly in individuals with the minor allele of the MTHFR missense SNP rs1801133. By elucidating the complex mechanism of the regulation of DNA methylation by both acquired and genetic factors, our results may be important for cancer prevention.

**Electronic supplementary material:**

The online version of this article (10.1186/s12885-018-4089-z) contains supplementary material, which is available to authorized users.

## Background

Aberrant DNA methylation is known as an essential factor for tumor initiation and promotion. Tumor cell genomes are generally characterized with region-specific hypermethylation and global hypomethylation; typically, the former occurs in promoter regions of tumor suppressor genes, and the latter is represented by hypomethylation in repetitive elements, such as LINE-1 [1–3]. In addition, several studies have reported an association between the level of global methylation of the peripheral blood leukocytes and the development of cancers, which suggests that metabolic conditions in cancer patients may induce aberrant DNA methylation systemically, not only in the primary organ [4–7]. Researchers in the epigenetics field conventionally consider aging and inflammation as initiators of aberrant DNA methylation [2, 8]. Methylation levels of many genes in healthy tissues alter with age [8–12], and aging is obviously related to tumor incidence, as a reflection of the mixture of complex exposures. Moreover, higher tumor incidence is also associated with various chronic inflammatory conditions, such as *H. pylori* (HP) gastritis and ulcerative colitis (UC), and previous reports suggest that the mechanisms of these associations can be through epigenetic alterations [8, 13]. In a colitis-induced mouse model, DNA hypermethylation was induced in low CpG regions called “DNA methylation valleys” [14].

DNA methylation is also known for its relationship with nutrition. In particular, one-carbon metabolism, which is tightly linked to folate metabolism, involves the step in which the methyl group is added to the cytosine base [15]. Our group previously observed a correlation between folate intake and methylation levels, and assessed interaction between SNPs related to one-carbon metabolism (such as MTHFR etc.) and its intake in Japanese healthy women [15]. In that study, we measured global DNA methylation levels of peripheral blood cells by the LUMA method [16, 17]. Although local tissues can be used to evaluate the effect of local (and mostly direct) exposure to risk factors (such as smoking, alcohol and infection) on DNA methylation, we used peripheral blood cells to assess the influence of systemic exposure such as nutrition levels and the association with serum marker values. Of course, the significance of variation in the DNA methylation of peripheral blood cells for tumorigenesis is not as simple as methylation levels in local tissues, such as gastrointestinal mucosa; nevertheless, it is considered a reasonable method for measuring the systemic effect of an “epimutagen” [4–6, 15, 18, 19].

Here, we focused on one of the most-used quantitative inflammation markers, CRP, and global methylation levels in peripheral blood cells. Since CRP is considered a disease marker for various diseases, including malignant tumors [20–24], the epigenetic effect of high-CRP conditions over the long term is a reasonable research question. In addition, our investigation included statistical adjustment of lifestyle-related characteristics, including folate intake, and examined statistical interaction by five one-carbon metabolism related-SNPs in the MTHFR, MTR, and MTRR genes. The study population was a well-characterized control group for a breast cancer case-control study in Japan, and validated methods were used to measure all variables. We consider that this study will contribute to understanding the mechanism by which systemic inflammation is associated with global DNA methylation, and of effect modification by individual folate-related factors.

## Methods

### Study subjects and data collection

Subjects were the control group of a hospital-based case-control study for breast cancer conducted from May 2001 to September 2005 at four hospitals in Nagano Prefecture, Japan. Details of this study have been described previously [25, 26]. In this study, healthy female individuals were selected by medical check-up in either of four hospitals and confirmed to not have any cancer. Each subject was recruited as a control for a case matched by age (within 3 years) and residential area. After exclusion of subjects with extremely low or high total energy intake (< 500 or 4000 kcal, respectively) or those without available DNA samples, 384 healthy Japanese women were included in the study.

Participants answered a self-administered questionnaire that included questions on the following: demographic and anthropometric characteristics, smoking habit, family history of cancer, physical activity, medical history, and menstrual and reproductive history. Dietary habits were investigated using a 136-item semiquantitative food frequency questionnaire (FFQ) that was developed and validated in a Japanese population [27, 28]. Spearman’s correlation coefficients between energy-adjusted folate intakes estimated from the FFQ and from dietary records were 0.35–0.50. Participants donated blood samples at the time they returned the questionnaire. Details of these protocols have been described previously [15].

### Laboratory analysis

Plasma CRP concentrations were measured by a commercial laboratory (LSI Medience Corporation, Tokyo, Japan) using a latex-enhanced high-sensitivity assay on a BN II nephelometer (Dade Behring Marburg GmbH, Marburg, Germany). The intra-assay coefficient of variation from the quality control samples was 4.8% at 0.036 mg/dL.

Genomic DNA was extracted from the whole blood using a Qiagen FlexiGene DNA Kit (Qiagen, Hilden, Germany) according to the manufacturer’s protocol. Global DNA methylation was quantified by Luminometric Methylation Assay (LUMA) [16, 17], which was described and validated for assessing small differences in our in-house testing [15]. Briefly, after cleaving 300 nanograms of genomic DNA with either HapII + EcoRI or MspI + EcoRI in two separate reactions, the HpaII/EcoRI and MspI/EcoRI ratios were calculated by pyrosequencing-based quantification of (dGTP + dCTP)/dATP for each reaction. The HpaII/MspI ratio was then calculated as (HpaII/EcoRI)/(MspI/EcoRI), which corresponds to the proportion of unmethylated cytosine in CCGG sequences in the genome [15]. Reproducibility of the assay for global methylation level was considered high (Total assay CV, 6.4%, which is always higher than intra- and inter- assay CV), suggesting that measurement errors were minimal.

In addition we focused on three genes, MTHFR, MTR, and MTRR, which are known to be involved in DNA methylation in one-carbon metabolism, and selected SNPs in consideration of the availability of functional information. Five polymorphisms in MTHFR (rs1801133 and rs1801131), MTR (rs1805087), and MTRR (rs10380 and rs162049) genes were genotyped by TaqMan SNP Genotyping Assay (Applied Biosystems, USA). These protocols have been described in detail elsewhere [15].

### Statistical analysis

#### Pre-analysis data processing

Folate intake was adjusted for total energy intake using the residual method. Serum CRP concentration and folate intake was divided into quartile categories because inappropriate influence of outlier should be avoided and linear correlation could not be assumed for untransformed value.

#### Adjustment for potential confounders

The following variables were used for adjustment: age (continuous); body mass index (BMI, continuous); smoking (never smokers, past smokers, current smokers); alcohol drinking (non-drinkers, occasional drinkers, regular drinkers of < 150 g ethanol/week, regular drinkers of ≥150 g ethanol/week); and physical activity in the past 5 years (no, 2 days/week, 3 days/week). Adjusted mean global methylation levels were calculated according to CRP categories and SNPs related to one-carbon metabolism using a multivariate linear regression model with covariates above. Subjects missing measurement data for at least one covariate were excluded from the multivariate analyses.

#### Testing for linear trend and statistical interaction

To test linear trends for mean CRP levels, regression coefficients (β) were calculated in the multivariable linear regression model using categories of each CRP level as ordinal variables. Log-transformed CRP was used in the model as a sensitivity analysis of linearity. To investigate statistical interaction by effect modifiers, subgroup analyses and tests for interaction (Wald test) were carried out with SNPs related to one-carbon metabolism and folate intake.

#### General considerations

Since the analyzed results are highly correlated with each other and considered to be too strict for exploratory research, statistical multiplicity was not adjusted. All *P*-values are two-sided, and those under 0.05 were considered significant. All statistical analyses were performed with SAS version 9.3 (SAS Institute, USA).

## Results

The study population is the same as in our previous study of DNA methylation and folate-related factors [15]. Briefly, mean age was 54.1 years (SD 10.3); mean body mass index was 23.0 (SD 3.2); smoking status was never 92.7%, former 2.1%, and current 5.2%; and drinking status was non-drinker 60.4%, sometimes 10.2%, < 150 g/week 22.7%, and ≥150 g/week 6.8%. After the evaluation of CRP distribution, we excluded four subjects with CRP > 0.5 mg/dL, which is an upper measurement limit of hs-CRP, because the possibility of acute inflammation could not be excluded. A total of 380 subjects was then used for analysis.

First, we investigated the crude association between serum CRP concentration and global DNA methylation (Table 1), but did not observe significant association (*P* for trend = 0.071). Next, we included potential confounders into the model. On adjustment for conventional lifestyle-related factors such as age, BMI, smoking, exercise, and drinking status, global methylation level was shown to be significantly increased by 0.43% per quartile category for CRP, and a trend test was statistically significant (adjusted model 1, *P* for trend = 0.011). Adjustment for BMI was considered to have the greatest influence on the coefficient of CRP (the coefficient changed from 0.28% to 0.42%, with *P* = 0.010 before/after BMI adjustment only). In addition, since we observed an association between folate intake and global methylation level [15], we included folate intake into the model (Adjusted Model 2), but the CRP trend did not remarkably change from the previous model (*P* for trend = 0.010). As shown in Table 1, log-transformed CRP (a numerical, not a categorical variable) was associated with global methylation level, and total energy intake did not influence the association (*P* for trend = 0.011). The association was still observed (*P* for trend = 0.019) and at the upper limit of total energy intake of 3000 kcal. Details of models are shown in Additional file 1: Table S1.

**Table 1:** Assessment of the association between serum CRP concentration and global methylation level with and without adjustment for life-style factors using the general linear model

				95% confidence interval			95% confidence interval		
Variable	CRP (mg/dL)	N	Estimated mean methylation (%)	Lower	Upper	β^c^	Lower	Upper	*P* value for trend
Crude model	0.002–0.011	101	69.7	69.0	70.3				
	0.012–0.023	89	70.2	69.5	70.9				
	0.024–0.048	93	70.7	70.0	71.3				
	0.049–0.491	93	70.4	69.8	71.1				
						0.28	−0.02	0.58	0.071
	Log-transformed CRP					0.25	−0.07	0.56	0.123
Adjusted model 1^a^	0.002–0.011	100	70.1	69.0	71.2				
	0.012–0.023	89	70.7	69.6	71.9				
	0.024–0.048	92	71.4	70.3	72.6				
	0.049–0.491	92	71.3	70.1	72.5				
						0.43	0.10	0.76	0.011
	Log-transformed CRP					0.42	0.07	0.78	0.019
Adjusted model 2^b^	0.002–0.011	100	70.0	68.9	71.1				
	0.012–0.023	89	70.8	69.6	71.9				
	0.024–0.048	92	71.4	70.2	72.6				
	0.049–0.491	92	71.3	70.1	72.4				
						0.43	0.10	0.76	0.010
	Log-transformed CRP					0.42	0.06	0.79	0.021

^a^Adjusted by age, BMI, smoking (never, former, current), exercise (none/week, 1–2 times/week, ≥3 times/week), and drinking (non-drinker, sometimes, < 150 g/week, ≥150 g/week). Estimates were calculated with average values of continuous variables

^b^Adjusted by the factors in adjusted model 1 with folate intake

^c^Average relative methylation difference per 1-category increase (included as a continuous variable)

Subsequently, we examined the statistical interaction by one-carbon metabolism related-SNP, folate intake and drinking status. As shown in Table 2, interaction between a polymorphism of MTHFR (rs1801133, known as C677T) and CRP was statistically significant (*P* for interaction = 0.046): the effect of CRP trend was significant in the CT/TT group (individuals with the minor allele T, *P* = 0.001), but no association was observed in the CC group (wild type). Although not presented in the table, the interaction was not significant (*P* = 0.109) when log-transformed CR*P* values were used instead of the CRP category, but the association was similar in the stratified analyses (β = 0.567, *P* = 0.005 in the CT/TT group; β = − 0.028, *P* = 0.932 in the CC group). Although no significant interaction was observed between the other SNPs and CRP status, one side of the divided subgroups showed significant *P* values in every analysis. Using Adjusted Model 2 in Table 1, we calculated adjusted estimates of global methylation levels stratified by CRP category. As shown in Table 3, a monotonous increase with CRP category was clearly observed in the MTHFR CT/TT group, but not in the CC group. In addition, on stratification all of subgroups by the single factors listed in Table 3, alcohol drinkers in the CT/TT group showed the highest regression coefficient (β = 0.83, *P* = 0.007) for the association between DNA methylation and CRP level (Table 4). A similar tendency was observed when using log-transformed CRP values.

**Table 2:** Assessment of interaction between serum CRP concentration and folate-related SNP/factors for global methylation levels

			95% confidence interval		*P* value for trend*	*P* for interaction*
	N	β*^c^	Lower	Upper		
MTHFR (rs1801133)						0.046
CT/TT	264	0.61	0.24	0.99	0.001	
CC	109	−0.09	−0.69	0.52	0.778	
MTHFR (rs1801131)						0.735
CC/CA	127	0.36	−0.17	0.89	0.186	
AA	246	0.47	0.07	0.87	0.022	
MTR (rs1805087)						0.627
GG/GA	120	0.34	−0.19	0.86	0.206	
AA	250	0.49	0.09	0.89	0.016	
MTRR (rs162049)						0.599
AA/AG	261	0.48	0.10	0.86	0.013	
GG	110	0.30	−0.30	0.89	0.328	
MTRR (rs10380)						0.423
TT/CT	79	0.66	0.02	1.30	0.043	
CC	292	0.37	−0.01	0.74	0.054	
Drinking status^a^						0.473
Non-drinker	223	0.33	−0.08	0.74	0.118	
Former/Current	150	0.56	0.06	1.06	0.028	
Folate intake^b^						0.736
< 340 μg/day	188	0.49	0.03	0.94	0.036	
≥340 μg/day	185	0.38	−0.06	0.83	0.092	

*Adjusted by age, BMI, smoking (never, former, current), exercise (none/week, 1–2 times/week, ≥3 times/week), drinking (non-drinker, sometimes, < 150 g/week, ≥150 g/week), and folate intake (quartile)

^a^ Adjusted by age, BMI, smoking (never, former, current), exercise (none/week, 1–2 times/week, ≥3 times/week), and folate intake (quartile)

^b^ Adjusted by age, BMI, smoking (never, former, current), exercise (none/week, 1–2 times/week, ≥3 times/week), and drinking (non-drinker, sometimes, < 150 g/week, ≥150 g/week)

^c^ Average relative methylation difference per 1-category increase (included as continuous variable)

**Table 3:** Adjusted estimates for mean methylation level stratified by quartile of serum CRP concentration

				95% confidence interval	
MTHFR (rs1801133)	CRP (mg/dL)	N	Estimated mean methylation (%)^a^	Lower	Upper
CT/TT	0.002–0.011	73	69.8	68.7	71.0
	0.012–0.023	59	70.5	69.2	71.7
	0.024–0.048	63	71.4	70.1	72.7
	0.049–0.491	69	71.6	70.4	72.8
CC	0.002–0.011	27	70.4	68.9	72.0
	0.012–0.023	30	71.3	69.8	72.8
	0.024–0.048	29	71.3	69.8	72.8
	0.049–0.491	23	70.1	68.4	71.8

^a^Adjusted by age, BMI, smoking (never, former, current), exercise (none/week, 1–2 times/week, ≥3 times/week), drinking (non-drinker, sometimes, < 150 g/week, ≥150 g/week), and folate intake (quartile). Estimates were calculated with average values of continuous variables

**Table 4:** Assessment of interaction between serum CRP concentration and drinking status in global methylation levels in the MTHFR CT/TT group

					95% confidence interval			95% confidence interval		
MTHFR (rs1801133)	Drinking status	CRP (mg/dL)	N	Estimated mean methylation (%)^a^	Lower	Upper	β^a^^b^	Lower	Upper	*P* value for trend^a^
CT/TT	Never	0.002–0.011	43	69.5	67.9	71.0				
		0.012–0.023	33	70.5	68.9	72.2				
		0.024–0.048	43	71.2	69.7	72.7				
		0.049–0.491	45	70.6	69.0	72.1				
							0.41	−0.07	0.90	0.094
		Log-transformed CRP					0.28	−0.25	0.81	0.307
	Former/Current	0.002–0.011	30	70.4	68.9	71.9				
		0.012–0.023	26	70.3	68.6	72.0				
		0.024–0.048	20	71.2	69.3	73.1				
		0.049–0.491	24	72.8	71.1	74.6				
							0.83	0.23	1.44	0.007
		Log-transformed CRP					0.88	0.26	1.50	0.006

^a^Adjusted by age, BMI, smoking (never, former, current), exercise (none/week, 1–2 times/week, ≥3 times/week), folate intake (quartiles). Estimates were calculated with average values of continuous variables

^b^Average relative methylation difference per 1-category increase (included as continuous variable)

**Fig. 1:**
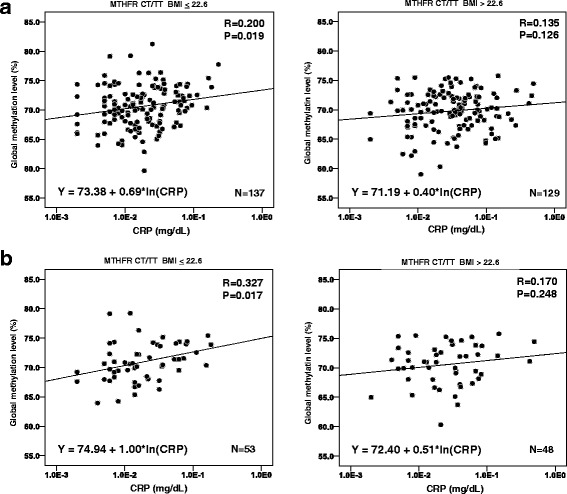
**a** Scatter plots for the correlation between CRP and global methylation in the CT/TT group stratified by the median of BMI. The scale of CRP was log-transformed. Formulas are calculated by single regression with natural logarithm of CRP and intercept. **b** Scatter plots for the correlation between CRP and global methylation in the CT/TT and former/current drinker group stratified by the median of BMI

Graphical representation of the association of CRP and global methylation is shown in scatter plots (Fig. 1a) of the results in the CT/TT group stratified by BMI, which is potentially the strongest confounder in the data analysis summarized in Table 1 and Additional file 1: Table S1. Removing individual covariates from the model produced the greatest change in the CRP correlation coefficient after removing BMI. As shown in Fig. 1, a weakly positive correlation was observed for the MTHFR CT/TT and BMI < 22.6. The cutoff was the median BMI. Correlation coefficients for CRP were similar those shown above. When the subjects were limited to the former/current drinkers only, the correlation was stronger (Fig. 1b). Additionally, we also measured serum HP antibody and pepsinogen levels, which directly reflect current/former chronic inflammation of the gastric mucosa due to infection, and investigated their association with global methylation levels with and without adjustment by CRP concentration. Results are summarized in Additional file 2: Table S2. Briefly, HP positivity (defined by an antibody level ≥ 10 U/ml) and pepsinogen positivity (defined by a pepsinogen I level < 70 ng/mL and pepsinogen I/II ratios ≤3.0 U/ml) were not significantly associated with global methylation either with or without adjustment. CRP was still associated with global methylation even with these variables (*P* = 0.009).

## Discussion

The study results show that serum CRP concentration was positively correlated with the global DNA methylation level of peripheral blood leukocytes. As mentioned in the introduction, it is widely known that chronic inflammation caused by infection or other factors is associated with aberrant DNA methylation, followed by cancer development [8, 13]. With respect to gastrointestinal inflammation, a potential association reported is that of the activation of reactive oxygen/nitrogen species with an increase in the expression of DNA methyltransferase (DNMT) [29, 30]. However, as DNA methylation increased by only 0.43% per quartile category for CRP in this study, the association should be considered weak even though it was significant. Few studies have assessed the association of methylation of peripheral leukocytes and CRP, but they reported no association between global LINE-1 methylation level and CRP [31, 32]. Thus, our finding needs to be interpreted with caution, especially because they can be explained by differences in white blood cell population [33]. Nevertheless, the SNP-and alcohol-associated interactions shown in Tables 3 and 4 indicate that the association we observed cannot be explained by these differences only.

Interestingly, we found that MTHFR genotype appears to modify the statistical association of CRP with global DNA methylation. The missense rs1801133 polymorphism (p.Ala222Val) in the MTHFR gene is known to affect enzyme activity, and its heterozygote CT and homozygotes TT have only around 65% and 30% of normal activity, respectively [34]. The loss of activity of MTHFR can lead to lower DNA methylation level through a dysfunction of conversion of 5,10-methylenetetrahydrofolate (5,10-MTHF) to 5-methyltetorahydrofolate (5-MTHF), which is a methyl donor for DNA methylation [35]. In a previous study, the MTHFR TT homozygote showed a lower methylation level when plasma folate level was low, whereas a significant positive correlation was not observed in the wild-type group (CC homozygote) [19]. That report also showed that MTHFR wild-type individuals maintain high methylation levels no matter how low plasma folate levels are. These findings suggested that variation in MTHFR activities can augment the variation of DNA methylation induced by other DNA methylation determinants (“epimutagens”). In our previous study, alcohol intake modified the association between folate intake level and global methylation level [15]. Alcohol consumption interferes with folate metabolism, which in turn disturbs methyl DNA synthesis. This suggests that modification of folate metabolism varies the regulation of DNA methylation by other epigenetically effective factors. With respect to the influence of alcohol, although there are some reports stating that excessive intake of alcohol is associated with peripheral blood global hypermethylation [36, 37], we did not observe a clear association between these aspects in our studies (15 and this study).

Regarding an association with diseases, a decrease in MTHFR activity does not necessarily facilitate cancer development. Zhou et al. reported that the homozygote of the minor allele of the rs1801133 polymorphism of the MTHFR (TT) gene was associated with reduced colorectal cancer risk [38], and low folate and high alcohol intake was more harmful for colorectal cancer incidence in the MTHFR wild type (CC) group than in TT group [39–44]. On the contrary, TT mutation was reported to be a risk factor for breast cancer incidence, and the association was strengthened by low folate intake or high alcohol intake [45–50]. In our present study, the MTHFR CT/TT group showed a stronger association between DNA methylation and CRP level than the other groups, particularly with alcohol consumption (Tables 2, 3 and 4). This suggests that different regulation patterns of DNA methylation (determined by MTHFR mutation, inflammation, alcohol consumption, folate intake etc.) are associated with different risks of disease. Although regulation of DNA methylation and its consequences are not monotonous, these past and our present findings suggest that the effects of potential epimutagens differ among individuals depending on genetic and other environmental status.

In addition, BMI had a strong influence as a confounding factor in this study. The background pertaining to confounding by BMI is considered as follows: there is an intermediate correlation between BMI and CRP, with the coefficient R being 0.265 (*P* < 0.001), and BMI is negatively correlated with global methylation, as shown in Additional file 1: Table S1. There are some reports stating that global DNA methylation is negatively correlated with numerical indicators of obesity, including BMI; this is consistent with our findings [51, 52].

Limitations of this study are as follows. First, since the study was conducted under a cross-sectional design, we cannot elucidate the causality of the observed associations. Second, although serum CRP concentration is an established predictive factor for many kind of diseases, including cancer and cardiovascular diseases [21–24, 53], particularly when assayed by the high-sensitivity method (see Materials and Methods), it is easily changed by acute infection or other cause of inflammation. To avoid misclassification and weakened associations, serum CRP concentration should ideally be measured in the steady state. Third, repeated comparisons for multiple subgroups were performed, which might have led to over-fitted and false-positive results by chance. Several associations we observed can be considered weak in spite of their statistical significance. As we did not investigate white blood cell population, change with systemic inflammation cannot be excluded. Finally, sample size was limited, and the study lacks sufficient statistical power to detect small effects and statistical interaction.

## Conclusion

Overall, our study suggests that systemic inflammation suggested by higher serum CRP is weakly associated with the global DNA hypermethylation of peripheral blood leukocytes. However, this association was more clearly seen among individuals carrying the minor allele of the MTHFR rs1801133 missense SNP (known as C677T), which has been shown to have induce reduced enzymatic activity and to cause global hypomethylation when folate level is low. Our results and previous findings also suggest that global DNA methylation is determined by many factors, including folate level, degree of systemic inflammation, metabolic status, and food and beverages intake, and that these items interact in a complex way. A comprehensive understanding of these interactions between acquired and genetic factors is critical to the realization of “tailor-made cancer prevention”.

## Additional files

Additional file 1: **Table S1.** Details of the models for the assessment of CRP and global methylation in all subjects. (DOCX 19 kb)

Additional file 2: **Table S2.** Assessment of association between HP infection and global methylation levels with/without adjustment with life-style factors, folate intake and serum CRP concentration. (DOCX 20 kb)

## Abbreviations

BMI: Body mass index
CRP: C-reactive-protein
HP: *H. pylori*
LUMA: Luminometric Methylation Assay
