# Supplementary material for: Correlation between global methylation level of peripheral blood leukocytes and serum C reactive protein level modified by MTHFR polymorphism: a cross-sectional study
Source: BMC Cancer. 2018 Feb 13;18:184. doi: 10.1186/s12885-018-4089-z (PMC5812223; doi:10.1186/s12885-018-4089-z)
Supplement: Supplementary file 1 — Table S1. Details of the models for the assessment of CRP and global methylation in all subjects. (DOCX 19 kb) [file 12885_2018_4089_MOESM1_ESM.docx]

| Supple. Table 1. Details of the models for the assessment of CRP and global methylation in all subjects. | | | | | | | | | | | | | | |  |  |  |  |  |  |
| --- | --- | --- | --- | --- | --- | --- | --- | --- | --- | --- | --- | --- | --- | --- | --- | --- | --- | --- | --- | --- |
|  |  |  |  |  |  |  |  |  |  |  |  |  |  |  |  |  |  |  |  |  |
|  |  | Crude model | | | |  | Adjusted model 1* | | | |  | Adjusted model 2† | | | |  | Adjusted model 3‡ | | | |
|  |  |  | 95%CI | |  |  |  | 95%CI | |  |  |  | 95%CI | |  |  |  | 95%CI | |  |
| Variables | Level | β | Lower | Upper | *P* value |  | β | Lower | Upper | *P* value |  | β | Lower | Upper | *P* value |  | β | Lower | Upper | *P* value |
| Intercept |  | 69.55 | 68.74 | 70.37 | <.001 |  | 71.78 | 68.36 | 75.21 | <.001 |  | 72.21 | 68.77 | 75.66 | <.001 |  | 74.22 | 70.56 | 77.87 | <.001 |
| CRP category |  | 0.28 | -0.02 | 0.58 | 0.071 |  | 0.43 | 0.10 | 0.76 | 0.011 |  | 0.43 | 0.10 | 0.76 | 0.010 |  | 0.42 | 0.10 | 0.75 | 0.011 |
| Age |  |  |  |  |  |  | 0.02 | -0.02 | 0.05 | 0.369 |  | 0.03 | -0.01 | 0.06 | 0.156 |  | 0.03 | -0.01 | 0.06 | 0.173 |
| BMI |  |  |  |  |  |  | -0.14 | -0.26 | -0.02 | 0.021 |  | -0.15 | -0.27 | -0.03 | 0.012 |  | -0.13 | -0.25 | -0.02 | 0.026 |
| Smoking | Never |  |  |  |  |  | -0.64 | -2.28 | 0.99 | 0.450 |  | -0.49 | -2.13 | 1.15 | 0.556 |  | -0.47 | -2.09 | 1.15 | 0.569 |
|  | Former |  |  |  |  |  | -0.03 | -2.88 | 2.82 | 0.991 |  | 0.11 | -2.73 | 2.96 | 0.937 |  | -0.10 | -2.91 | 2.72 | 0.945 |
|  | Current |  |  |  |  |  | Ref. |  |  | . |  | Ref. |  |  |  |  | Ref. |  |  |  |
| Exercise | 0/week |  |  |  |  |  | -0.39 | -1.38 | 0.60 | 0.381 |  | -0.49 | -1.48 | 0.51 | 0.335 |  | -0.52 | -1.50 | 0.46 | 0.299 |
|  | 1-2/week |  |  |  |  |  | 0.25 | -0.85 | 1.36 | 0.684 |  | 0.24 | -0.86 | 1.34 | 0.667 |  | 0.12 | -0.97 | 1.21 | 0.824 |
|  | 3</week |  |  |  |  |  | Ref. |  |  | . |  | Ref. |  |  |  |  | Ref. |  |  |  |
| Drinking | Never |  |  |  |  |  | 0.19 | -1.23 | 1.60 | 0.817 |  | 0.17 | -1.23 | 1.58 | 0.808 |  | -0.26 | -1.69 | 1.16 | 0.715 |
|  | Sometimes |  |  |  |  |  | 1.05 | -0.67 | 2.77 | 0.237 |  | 1.02 | -0.69 | 2.74 | 0.242 |  | 0.54 | -1.19 | 2.27 | 0.542 |
|  | <150g/week |  |  |  |  |  | 0.97 | -0.58 | 2.52 | 0.250 |  | 0.99 | -0.55 | 2.53 | 0.207 |  | 0.58 | -0.97 | 2.13 | 0.464 |
|  | 150<g/week |  |  |  |  |  | Ref. |  |  | . |  | Ref. |  |  |  |  | Ref. |  |  |  |
| Folate intake |  |  |  |  |  |  |  |  |  |  |  | -0.32 | -0.65 | 0.00 | 0.052 |  | -0.32 | -0.64 | 0.00 | 0.052 |
| Total energy |  |  |  |  |  |  |  |  |  |  |  |  |  |  |  |  | -0.001 | -0.002 | 0.000 | 0.003 |
| * Adjusted by age, BMI, smoking (never, former, current), exercise (none/week, 1-2 times/week, ≥3 times/week), and drinking (non-drinker, sometimes, <150 g/week, ≥150 g/week). Estimates were calculated with average values of continuous variables. | | | | | | | | | | | | | | | | | | | | |
|  |  |  |  |  |  |  |  |  |  |  |  |  |  |  |  |  |  |  |  |  |
| † Adjusted by the factors in adjusted model 1 with folate intake | | | | | | | | | | | | | | | | | | | | |
| ‡ Adjusted by the factors in adjusted model 1 with folate intake and total energy intake | | | | | | | | | |  |  |  |  |  |  |  |  |  |  |  |
