# Supplementary material for: Correlation between global methylation level of peripheral blood leukocytes and serum C reactive protein level modified by MTHFR polymorphism: a cross-sectional study
Source: BMC Cancer. 2018 Feb 13;18:184. doi: 10.1186/s12885-018-4089-z (PMC5812223; doi:10.1186/s12885-018-4089-z)
Supplement: Supplementary file 2 — Table S2. Assessment of association between HP infection and global methylation levels with/without adjustment with life-style factors, folate intake and serum CRP concentration. (DOCX 20 kb) [file 12885_2018_4089_MOESM2_ESM.docx]

| Supplementary Table 2. Assessment of association between HP infection and global methylation levels with/without adjustment with life-style factors, folate intake and serum CRP concentration | | | | | | | |
| --- | --- | --- | --- | --- | --- | --- | --- |
|  |  |  |  |  |  |  |  |
|  |  |  |  |  | 95% confidence interval | |  |
| Variable | Factors |  | N | Estimated mean methylation  (%) | Lower | Upper | *P* value |
| Crude model | H. pyroli antibody | - | 213 | 70.6 | 69.6 | 71.6 | 0.260 |
|  |  | + | 167 | 71.0 | 69.9 | 72.1 |  |
|  | Pepsinogen | - | 286 | 70.0 | 69.6 | 70.5 | 0.425 |
|  |  | + | 94 | 70.4 | 69.9 | 70.9 |  |
| Adjusted model 1* | H. pyroli antibody | - | 213 | 70.6 | 69.6 | 71.6 | 0.302 |
|  |  | + | 167 | 71.0 | 69.9 | 72.1 |  |
|  | Pepsinogen | - | 286 | 70.7 | 69.7 | 71.7 | 0.366 |
|  |  | + | 94 | 71.1 | 69.9 | 72.3 |  |
| Adjusted model 2† | H. pyroli antibody | - | 213 | 70.7 | 69.7 | 71.7 | 0.269 |
|  |  | + | 167 | 71.1 | 70.0 | 72.2 |  |
|  | Pepsinogen | - | 286 | 70.8 | 69.8 | 71.7 | 0.310 |
|  |  | + | 94 | 71.2 | 70.0 | 72.4 |  |
| * Adjusted by age, BMI, smoking (never, former, current), exercise (none/week, 1-2 times/week, ≥3 times/week), drinking (non-drinker, sometimes, <150 g/week, 150< g/week), and folate intake (quartile). Estimates were calculated with average values of continuous variables. | | | | | | | |
| † Adjusted by the factors in adjusted model 1 with CRP (quartile). Estimates were calculated with average values of continuous variables. | | | | | | | |
